# miR-300 mediates Bmi1 function and regulates differentiation in primitive cardiac progenitors

**DOI:** 10.1038/cddis.2015.255

**Published:** 2015-10-29

**Authors:** F M Cruz, M Tomé, J A Bernal, A Bernad

**Affiliations:** 1 Department of Cardiovascular Development and Repair, Centro Nacional de Investigaciones Cardiovasculares (CNIC), Madrid, Spain; 2 Department of Immunology and Oncology, Spanish National Center for Biotechnology (CNB-CSIC), Campus Universidad Autónoma de Madrid, 28049 Madrid, Spain

## Abstract

B lymphoma Mo-MLV insertion region 1 (Bmi1) is a polycomb-family transcriptional factor critical for self-renewal in many adult stem cells and human neoplasia. We sought to identify microRNAs regulated by Bmi1 that could play a role in multipotent cardiac progenitor cell (CPC) decisions. We found that miR-300, a poorly characterized microRNA mapping in the *Dlk1-Dio3* microRNA cluster, was positively regulated by Bmi1 in CPCs. Forced expression of miR-300 in CPCs promoted an improved stemness signature with a significant increase in Oct4 levels, a reduction in senescence progression and an enhanced proliferative status via p19 activation and inhibition of p16 accumulation. Endothelial and cardiogenic differentiation were clearly compromised by sustained miR-300 expression. Additionally, RNA and protein analysis revealed a significant reduction in key cardiac transcription factors, including Nkx2.5 and Tbx5. Collectively, these results suggest that some functions attributed to Bmi1 are due to induction of miR-300, which decreases the cardiogenic differentiation potential of multipotent CPCs *in vitro* and promotes self-renewal.

Bmi1 (B lymphoma Mo-MLV insertion region 1), a member of polycomb repressive complex 1 (PCR1), is a transcription factor involved in multiple biological processes, including embryonic development, organ formation, tumorogenesis, and stem cell self-renewal and differentiation.^1^ Deficiency in Bmi1 results in progressive postnatal growth retardation and neurological defects, confirming that Bmi1 is required for the self-renewal of stem cells in the peripheral and central nervous systems, but not for their survival or differentiation.^2^ In the absence of Bmi1, the cyclin-dependent kinase inhibitor *p16Ink4a* is generally upregulated,^2, 3^ reducing the rate of cellular proliferation. *BMI1* is downregulated by replicative senescence in human fibroblasts and overexpression of *BMI1* extends replicative life span, correlating with *INK4A/ARF* deregulation.^4^ *Bmi1* has been reported to play crucial roles during the self-renewal and maintenance of hematopoietic, intestinal, bronchioalveolar, pancreatic, prostate, lung, and epithelial stem cells^2, 3, 4, 5, 6^ and more recently the tongue and rodent incisors.^7^ Thus, *Bmi1* is possibly the most universal adult stem cell marker and a remarkable player in many cancer stem cells models.^8, 9^ In all models tested, *Bmi1* dependence seems to distinguish stem cell self-renewal from committed progenitor proliferation.^2^

Multiple functions have been ascribed to *Bmi1*, underscoring its broad importance in numerous processes. *Bmi1* facilitates the repression of *Hox* genes to prevent inappropriate differentiation and induces downregulation of Dickopf (DKK) proteins, resulting in upregulation of the *Wnt* target *c-Myc*, which in turn leads to transcriptional autoactivation of *Bmi1*.^7^ Reinforcement of this positive feedback loop regulating *Bmi1* expression seems relevant in promoting cancer and maintaining the stem cell phenotype.^9, 10^ In addition, *Bmi1* is transcriptionally controlled by phosphorylated *Nanog*,^11, 12^ and is inhibited by a plethora of miRNAs that are significantly downregulated in cancer models.^12^

Extensive studies on primed differentiation of murine embryonic stem (ES) and adult stem cells have established that efficient stem cell maintenance requires a highly concerted regulation of gene expression,^13, 14^ involving both coding genes and noncoding RNAs (ncRNAs). Among the regulatory elements involved in stem cell function, Oct4 protein, encoded by the *Pou5f1* gene, is essential for the stemness properties of ES cells and is a key regulator of pluripotency in mammalian development. The Oct4 transcription factor is also indispensible for somatic cell reprogramming. Besides transcription factors, microRNAs (miRNAs) also play important roles in stem cell homeostasis. miRNAs are an abundant class of small ncRNAs that regulate the translation, stability, and localization of target messenger RNAs. Functional studies in ES cells have demonstrated critical roles for miRNAs, especially in regulating the balance between self-renewal and differentiation.^14, 15^ Less information is available about the role(s) of specific miRNAs in the regulation of adult stem cell systems, but some studies have explored possible collaborations; for example, miR-142-3p controls specification of definitive hemangioblasts during ontogeny.^16, 17^ Also, miR-21 strongly inhibits *SOX2* expression in human mesenchymal stem cells (MSCs), resulting in reduced clonogenic and proliferative potential and cell-cycle arrest.^18^ Finally, Lin28a, a highly conserved RNA-binding protein expressed during embryogenesis, plays important roles in development, pluripotency, and metabolism (reviewed in Shyh-Chang *et al.*^19^), and its expression in several adult injury models improves repair processes in a manner mediated by its inhibition of let-7 miRNA biogenesis.^19^ Regarding the involvement of miRNAs in *Bmi1* regulation, there are multiple descriptions of miRNAs targeting *Bmi1* and many of them impact the corresponding stem or cancer stem cell function (Supplementary Table S1).

Adult resident cardiac stem/progenitor cells (CSCs/CPCs) are implicated in homeostatic turnover of the adult heart (reviewed in Malliaras *et al.*^20^). Among the different populations of putative CSCs isolated to date (reviewed in Martin-Puig *et al.*^21^ Urbanek *et al.*^22^ and Li *et al.*^23^), those expressing surface markers such as c-kit, SCA1, ATP-binding cassette *Abcg2*, or *PDGFRα* are the principal candidates. Indeed, resident CPCs with genetic elimination of *Sca1* do not properly respond to pathological damage *in vivo* and show impaired growth and survival *in vitro*.^24^ In another study, Ellison *et al.*^25^ have proposed that cKIT+ CSCs are necessary and sufficient for adult heart turnover, although a recent lineage-tracing experiment questioned the relevance of cKIT+ CSCs to *in vivo* cardiomyocyte turnover.^26^ These authors claimed that, in all probability, diversity in CSC populations could be related to some heterogeneity within the CSC compartment.^25^ Nevertheless, two clinical evaluations have been initiated with promising results in early phases.^27, 28^

Here, using murine CPCs, we attempted to identify miRNAs that could be directly regulated by Bmi1. We hypothesized that those miRNAs upregulated in parallel with *Bmi1* could be involved in the maintenance of the undifferentiated state. Conversely, miRNAs downregulated with *Bmi1* might be involved in early commitment/differentiation decisions. We show that miR-300 is positively regulated in parallel with *Bmi1* expression in CPCs, while miR-188 and miR-362 are inversely regulated. Furthermore, we confirm that miR-300 is a target of Bmi1 and show that forced expression of miR-300 favors the undifferentiated state and impedes differentiation of CPCs. Indeed, CPCs engineered to overexpress miR-300 exhibit compromised spontaneous endothelial differentiation and show a poor response to cardiomyogenic stimuli, an outcome that is possibly mediated by severe inhibition of Nkx2.5 expression.

## Results

### Bmi1 regulates miR-300 expression in CPCs

**Figure 1:**
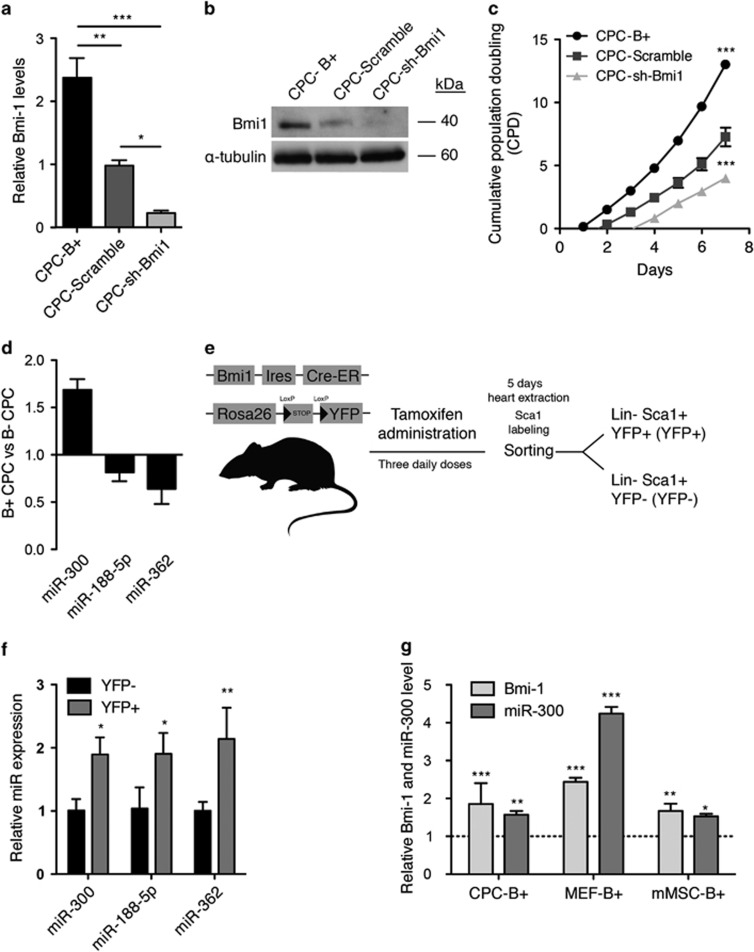
miR-300 expression is increased in Bmi1-overexpressing cells. CPCs were transduced with lentiviral vectors expressing Bmi1, sh-Scramble (encoding a negative control shRNA), and sh-Bmi1. Transduced cells were purified by FACS. Bmi1 expression was assessed in purified cells by (**a**) RT-qPCR and (**b**) western blot. (**c**) Cumulative population doubling (CPD) was estimated in CPC-B+ (closed circles), CPC-Scramble (gray squares), and CPC-sh-Bmi1 (triangles) cells. (**d**) RT-qPCR validation of the array results for miR-300, miR-188, and miR-362; miR-346 was not validated. (**e**) Isolation of an identified primary subpopulation of Sca1-CPCs characterized by moderate-high expression of Bmi1 using conditional Bmi1-YFP mice. (**f**) Comparison of miRNA expression in Sca1 compartments (YFP+ *versus* YFP−). (**g**) Relative expression of miR-300 in correlation with Bmi1 expression in cardiac progenitor cells (CPCs), mouse embryonic fibroblasts (MEFs), and mesenchymal stem cells (MSCs). ***P<0.001, **P<0.01, *P<0.05 (two-way ANOVA followed by Bonferroni post-test; means±S.E.M., *n*=5)

Freshly isolated Lin-SCA1+ CPCs (SCA1-CPCs) from heart are defined as *Cd45* negative and express a panel of multipotency-related genes, including *Tbx5, Klf4, c-Myc, Bmi1, and Meg3*, and a distinctive profile of endothelial, fibroblast, and cardiac markers (Supplementary Figure S1). To assess the potential involvement of *Bmi1* in the control of miRNA expression in SCA1-CPCs (hereafter named CPCs), we transduced freshly isolated CPCs with lentiviral vectors (Supplementary Figure S2a) engineered to overexpress or silence *Bmi1* (B+ and sh-Bmi1, respectively). We first confirmed *Bmi1* up- or downregulation in transduced cells by RT-qPCR and western blotting (Figures 1a and b). As expected, CPC-B+ cells grew significantly faster than equivalent cells expressing an shRNA against *Bmi1* or a scrambled shRNA control (Figure 1c). We then compared the respective miRNA expression profiles (Microarray v1.0) of B+ and sh-Bmi1 CPCs as described,^29^ and considered miRNAs that showed a significant upregulation in B+ CPCs and a reduction in sh-Bmi1 CPCs. From these results (GSE66813), we compiled a short list of candidate miRNAs showing *Bmi1*-dependent changes in expression (Supplementary Figure S3a). The expression profile of three miRNAs, miR-300, miR-188, and miR-362, was validated by RT-qPCR, but miR-346 was not confirmed (Figure 1d). The expression profile of the three confirmed miRNAs in CPCs corresponded to miR-362>miR-188>miR-300 (Supplementary Figure S3b). We next evaluated endogenous expression of these miRNAs in CPCs with moderate-high expression of *Bmi1*. To obtain this population, we marked *Bmi1*-expressing cells in the mouse and traced their descendants using an inducible *Bmi1 CreERT2* strain^30^ crossed to a Rosa26-floxed yellow fluorescent protein (YFP) reporter line. Induction of Cre recombination by tamoxifen results in permanent expression of YFP in *Bmi1*-expressing cells and their progeny (Figure 1e). Subsequently, we compared CPC-B+ cells labeled by YFP (YFP+) with the remaining CPC compartment (YFP−). Results confirmed an upregulation of miR-300 in the YFP+ (CPC-B+) population, but not miR-188 or miR-362, which were not overrepresented in the YFP− population (Figure 1f). We validated this result in other cell lines. Accordingly, overexpression of *Bmi1* in mouse embryonic fibroblasts (MEFs) and adipose tissue-derived MSCs induced the upregulation of miR-300 relative to control cells (GFP), although the relative increase was cell-type dependent (Figure 1g). Specifically, *Bmi1*-induced upregulation of miR-300 in CPCs (1.35-fold) was found to be similar to MSCs (1.5-fold), but lower than that observed in MEFs (4.3-fold). Taken together, these results suggest that *Bmi1* might directly or indirectly regulate miR-300 expression.

### miR-300 counteracts CPC replicative senescence

**Figure 2:**
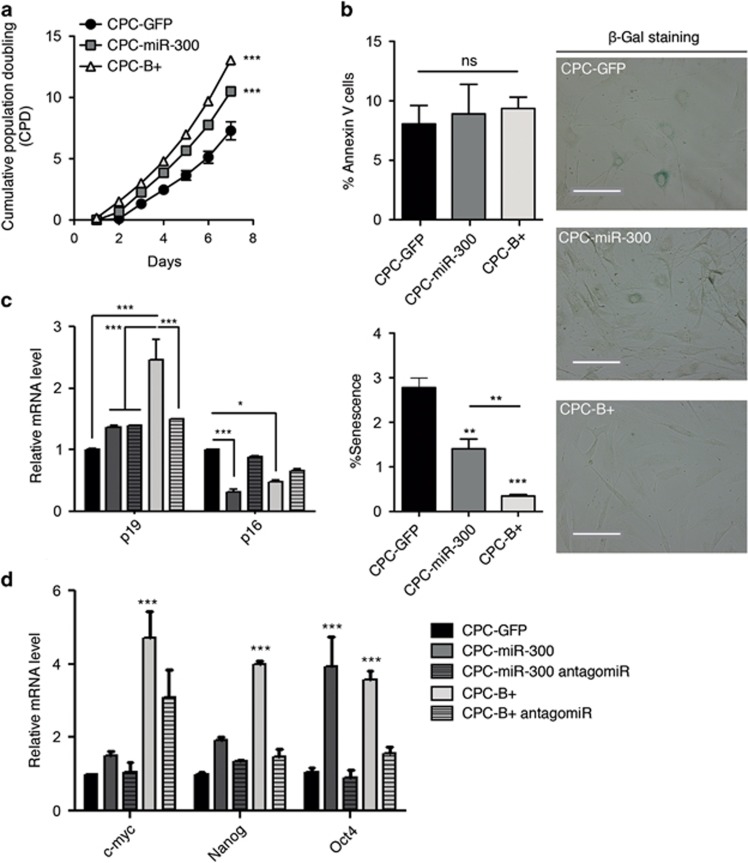
miR-300 delays CPC senescence. (**a**) Cumulative population doubling over several culture passages of CPC-GFP (closed circles), CPC-miR-300 (gray squares), and CPC-Bmi1(B+ triangles). (**b**) Apoptosis and senescence determined by annexin V staining (left upper panel) and *β*-gal staining (left lower panel), respectively; the right panels show representative *β*-gal staining images. Bars, 100 μm. (**c, d**) Panels of relative expression levels of *p19* and *p16* (**c**) and multipotency-related genes (**d**) in CPC-manipulated cells; where indicated, cells were transfected with mirVana miR-300 inhibitor. ***P<0.001; *P<0.05; ns, not significant (two-way ANOVA followed by Bonferroni post-test; means±S.E.M., *n*=5)

Because *Bmi1* upregulation increased cellular proliferation, we questioned whether this was mediated by miR-300. We therefore overexpressed miR-300 in CPCs using a non-viral piggyBac vector (Supplementary Figure S2b). CPC-miR-300 cells exhibited a ≈5-fold increase in miR-300 compared with control (CPC-GFP) cells, whereas CPC-B+ cells had a ≈1.7-fold increase (Supplementary Figure S3c). Growth rates of CPC-miR-300 and CPC-B+ cells, at all points analyzed, were significantly higher than CPC-GFP cells (Figure 2a). This increase in growth was not the result of changes in apoptosis since annexin V staining was equivalent in the three groups (Figure 2b, top left); however, significant differences were found in the proportion of senescent cells measured by *β*-galactosidase activity (Figure 2b, right panels). Accordingly, the number of *β*-Gal+ cells was reduced 2.2-fold in CPC-miR-300 and 6-fold in CPC-B+ populations compared with control (Figure 2b, bottom left), suggesting that miR-300 overexpression contributes to reduce and/or delay senescence. Consistent with a modification in senescence development, differences were found in cell-cycle distribution in miR-300-expressing cells (Supplementary Figure S4). Gene expression analysis of cell-cycle senescence-associated and stemness markers in CPC-miR-300 and CPC-B+ cells demonstrated a significant upregulation in *p19, c-myc, Nanog, and Oct4* levels and a clear reduction in *p16* levels (Figures 2c and d). To further examine whether miR-300 is a relevant downstream effector of Bmi1, we knocked down miR-300 expression in CPC-miR-300 and CPC-B+ cells using anti-miR-300 oligonucleotides. We titrated the anti-miR concentration required to achieve at least a 50% reduction of miR-300 expression in both cell lines (Supplementary Figure S5). Results showed that anti-miR-300 (50 pmol) suppressed the *Bmi1-*mediated effect on several target genes (Figures 2c and d). In particular, BMI1-induced *Oct4* expression was fully dependent on miR-300. Thus, knockdown of miR-300 in CPC-B+ and CPC-miR-300 cells reduced *Oct4* expression to basal levels compared with control cells. Furthermore, miR-300 overexpression induced an upregulation of *Oct4* mRNA to levels similar to those in CPC-B+ cells (Figures 2c and d). Collectively, these results suggest that miR-300 is necessary for *Bmi1*-dependent expression of genes involved in cell-cycle dynamics, which might preserve CPCs in a more undifferentiated state. Indeed, genes related to multipotency and stemness were found to be overrepresented in CPC-B+ and CPC-miR-300 cells (Figures 2c and d), and recovered to normal levels when miR-300 was downregulated. In summary, miR-300 enhances the expression of a panel of multipotent genes, indicating a plausible association with the maintenance of CPCs in a more undifferentiated state.

### miR-300 in the *Dlk1-Dio3* context

**Figure 3:**
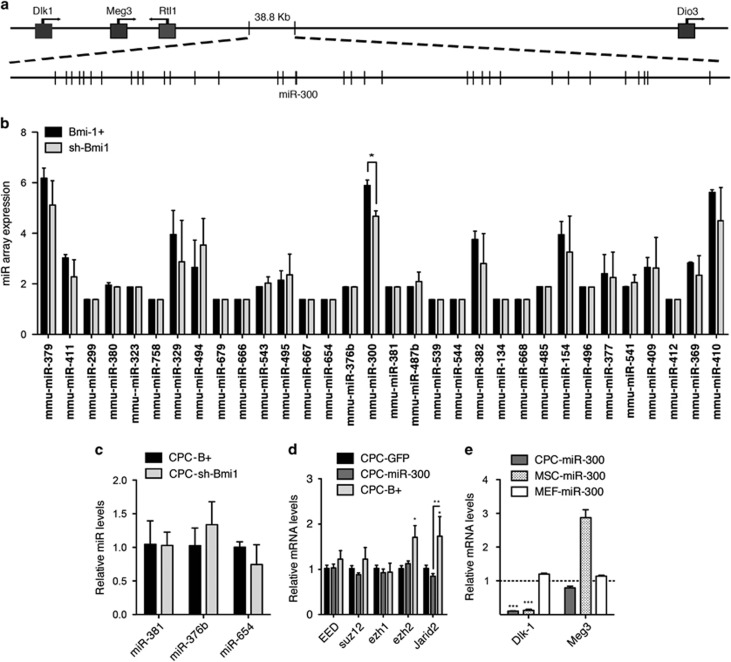
miR-300 belongs to the imprinted *Dlk1-Dio3* cluster. (**a**) Simplified map (5′→ 3′) of the mouse *DlK-1-Dio3* cluster (chromosome 12qF1); miR-300 is shown. (**b**) miR array results at the *DlK-1-Dio3* cluster. (**c**) Direct validation by RT-qPCR of miR-300 neighbors, miR-381, miR-376b, and miR-654, confirmed the specificity of association of Bmi1 and increased expression of miR-300. (**d**) RT-qPCR analysis of different polycomb repressor complex 1/2 members and recruiters in CPC-GFP, CPC-B+, and CPC-miR-300 cells. (**e**) RT-qPCR analysis of different *Dlk1-Dio3* cluster inductors in CPC-miR-300, MEF-miR-300, and MSC-miR-300 cells relative to their respective control (−GFP) cultures. ***P<0.001, **P<0.01, *P<0.05 (two-way ANOVA followed by Bonferroni post-test; means±S.E.M., *n*=5)

In humans, miR-300 localizes to a dense miRNA cluster residing in chromosome 14, region 14q32.31 (*DLK1-MEG3;* Figure 3a). In the mouse, this region corresponds to the *Dlk1/Gtl2* domain located on chromosome 12qF1.^31^ Detailed miRNA array expression analysis of the whole miRNA-encoding locus in CPC-B+ and CPC-sh-Bmi1 cells showed that miR-300 was the only miRNA significantly modulated by overexpression of Bmi1 (Figure 3b). This was confirmed by RT-qPCR analysis of neighboring miRNAs (Figure 3c). Because *DLK1-MEG3* regulation is dependent on polycomb-containing complexes,^28^ we assessed whether miR-300 could modulate members of the polycomb complexes PRC1 and PRC2. We found a modest decrease in the expression of *Suz12, Ezh1,* and *Jarid2* in CPC-miR-300 cells relative to CPC-GFP cultures (Figure 3d). In contrast, CPC-B+ cells displayed a different expression pattern, with a moderate increase in *Eed* and *Suz12* expression and a significant upregulation of *Ezh2* and *Jarid2* (Figure 3d). Additionally, we found that modulation of miR-300 expression also affected other elements of the *DLK1-MEG3* domain in different cell types (Figure 3e), which was contingent on Bmi1 regulation (Supplementary Figure S6). Accordingly, miR-300 overexpression provoked a decrease in *Dlk1* mRNA levels in CPCs and MSCs, but not in MEFs (Figure 3e), suggesting a context-dependent regulation. This effect was also observed for *Meg3*, which was reduced by miR-300 overexpression in CPCs, but was increased in MSCs (Figure 3e). Collectively, these data illustrate the great complexity of the regulatory mechanisms underlying gene expression at this locus (reviewed in Royo and Cavaille^32^), but also highlight the apparent selectivity of *Bmi1* for miR-300 expression and the consequent impact on some elements of the *DLK1-MEG3* domain.

### Forced expression of miR-300 impairs CPC differentiation

**Figure 4:**
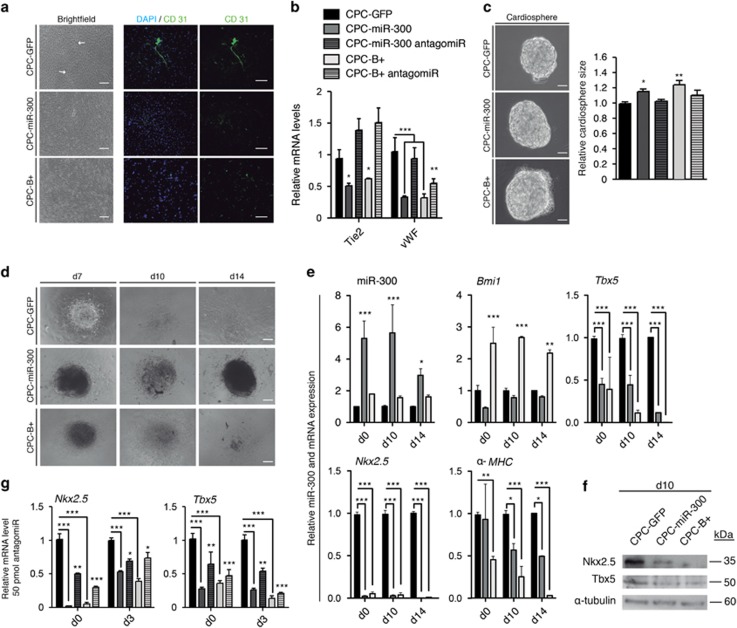
miR-300 diminishes CPC differentiation potential in cardiospheres. (**a**) Representative images of spontaneous endothelial differentiation in transduced cells after 10–12 days of differentiation. Arrows indicate tubular structures in brightfield, which were positive for CD31. Bars, 200 μm. (**b**) RT-qPCR analysis of endothelial genes *(Tie2* and *vWF)* in CPC-GFP (black bars), CPC-miR-300 (gray bars), and CPC-B+ (white bars) cells. (**c**) Representative cardiosphere images at day 6 of differentiation. Bars, 100 *μ*m (left). Relative cardiosphere size (right). (**d**) Cardiac differentiation induced by retinoic acid; representative images of transfected CPCs at day (d)7, d10, and d14 of the differentiation protocol. Bars, 200 μm. (**e**) Selected cardiac genes were measured by RT-qPCR in transfected cells during differentiation (d0, d10, and d14). (**f**) Western blotting of total protein extracts was performed at d10. (**g**) RT-qPCR analysis of selected cardiac genes in CPCs transfected with mirVana miR-300 inhibitor. ***P<0.001, **P<0.01, *P<0.05 (two-way ANOVA followed by Bonferroni post-test; means±S.E.M., *n*=6)

To test the role of miR-300 in CPC differentiation, cells were first allowed to spontaneously differentiate in culture and endothelial commitment was measured. CPC-GFP cells, but not CPC-miR-300 or CPC-B+ cells, formed reticular structures after 10–12 days (Figure 4a, brightfield), which were positive for CD31 (Figure 4a). Moreover, mRNA analysis of endothelial markers revealed that *Tie2* and *vWf* expression was significantly reduced in CPC-miR-300 and CPC-B+ cells compared with control CPC-GFP cells (Figure 4b). These data suggest a functional impairment of endothelial cell differentiation in miR-300 and *Bmi1*-expressing cells. To test the generality of this response, we used the same cell populations and induced cardiac differentiation by the hanging drop method. After 6 days in culture, cardiospheres from CPC-miR-300 and CPC-B+ cells were significantly larger than those formed from CPC-GFP cells (Figure 4c), corroborating the finding of increased proliferation potential (Figure 2a). Additionally, knockdown of miR-300 in CPC-miR-300 cells led to a significant reduction in cardiosphere size (Figure 4c, right panel). Following retinoic acid treatment to promote cardiac differentiation, plated CPC-B+ cardiospheres presented an apparently slower differentiation kinetic as observed by a reduction in cell spreading compared with control cells. Also, CPC-miR-300 cardiospheres continued to proliferate with minimal differentiation compared with CPC-GFP control cells, which showed greater expansion (Figure 4d). Time course analysis of cardiac marker gene expression revealed that, compared with control cells, levels of *α-MHC*, Nkx2.5, and Tbx5 were significantly reduced in CPC-B+ and CPC-miR-300 cells throughout the differentiation period (Figure 4e). Indeed, at d14 of differentiation, *α-MHC* gene expression was almost abolished in CPC-B+. Of note, *Nkx2.5* gene expression was scarcely detected at any period in CPC-B+ and CPC-miR-300 (Figure 4e), and this was confirmed by western blot analysis (Figure 4f). Because *Nkx2.5* is a critical cardiogenic transcription factor,^30^ its evident absence in these cells likely contributes to the impaired cardiogenic differentiation observed. Finally, to confirm that miR-300 is a downstream effector of *Bmi1* during the early steps of CPC cardiogenic differentiation, we analyzed *Nkx2.5* and *Tbx5* expression early after anti-miR-300 transfection. Exposure to anti-miR-300 for as little as 3 days resulted in a partial rescue of *Nkx2.5* and *Tbx5* expression in CPC-B+ and CPC-miR-300 cells compared with control cells (Figure 4g).

### miR-300/*Bmi1* interaction network in CPC

**Figure 5:**
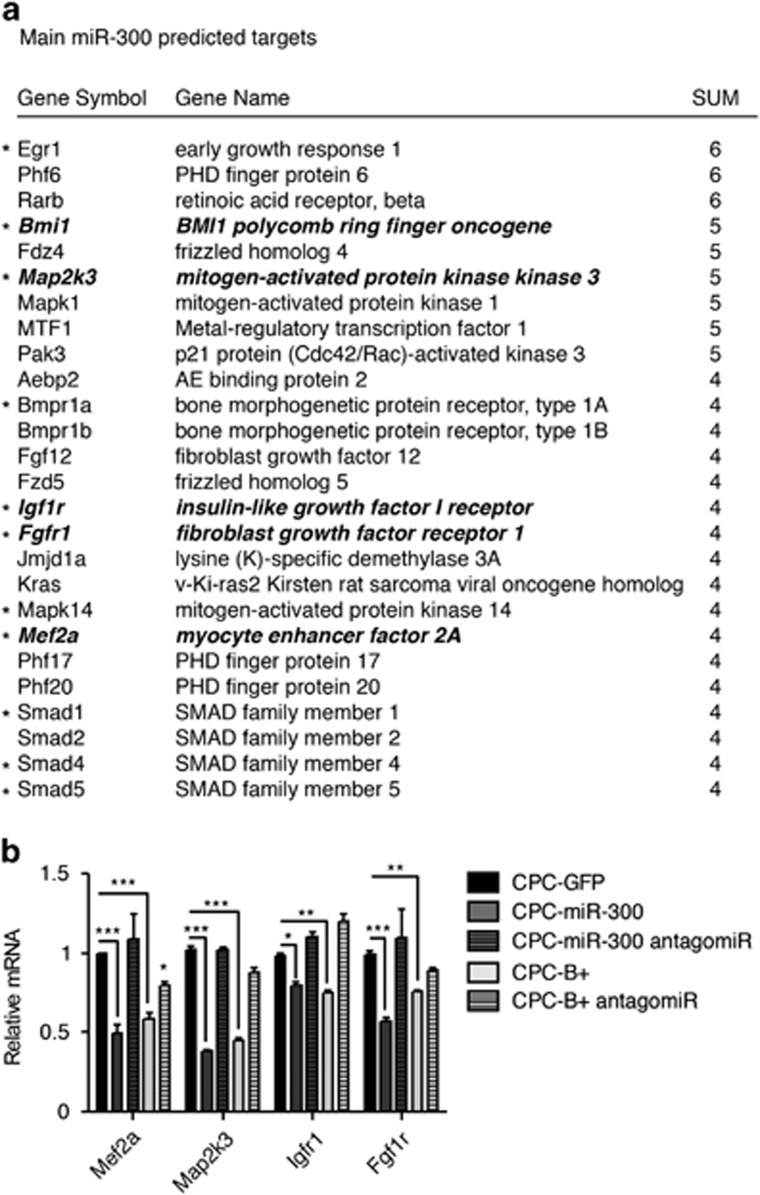
miR-300 regulatory potential. (**a**) Main plausible targets of miR-300 were identified by comparing all predicted miRNA target sites in miRWalk database produced by Diana, Pictar4, miRanda, Pita, TargetScan, and RNA22 databases, containing all the putative targets. Main miR-300 predicted targets with at least four hits in these databases (indicated in the table) were selected. Asterisks indicate tested targets (11 in total) and in bold-italic those validated targets. (**b**) RT-qPCR analysis of CPC; where indicated, cells were transfected with mirVana miR-300 inhibitor. ***P<0.001, **P<0.01, *P<0.05 (two-way ANOVA followed by Bonferroni post-test; means±S.E.M., *n*=5)

Little is known about the physiological roles of miR-300. We compared all potential miR-300 target genes predicted by specialized databases and Ingenuity Pathway Analysis (IPA) was used to link the direct targets. The most significant targets found by IPA are shown in Figure 5. These included genes in the *Tgf-β* signaling pathways (*Map2k3*) and factors involved in vertebrate cardiogenesis (*Smad2, Mapk14, Bmpr1a*) (Figure 5a). The *Tgf-β* pathway is associated with the regulation of cardiac differentiation.^33^ Another target, *Map2k3,* of the *Fgf* signaling pathway has been also described to be important incardiac differentiation^34^ and affects mainly the *p38 Mapk* pathway.^35, 36^ Eleven putative targets were selected for validation (Figure 5b and Supplementary Figure S6). RT-qPCR analysis revealed that *Mef2a, Map2k3, Igfr1, and Fgf1r* expression was dependent on miR-300 expression, and this decrease in expression could be reversed by transfection of an anti-miR-300 (five shown in bold in Figure 5a). A similar modulation was found in CPC-B+ cells (Figure 5b). Expression of the remaining putative miR-300 targets tested was not influenced by miR-300, although all were inhibited by *Bmi1* overexpression (Supplementary Figure S6). The finding of *Bmi1* as a highly probable target of miR-300 suggests that it could directly regulate *Bmi1*.

**Figure 6:**
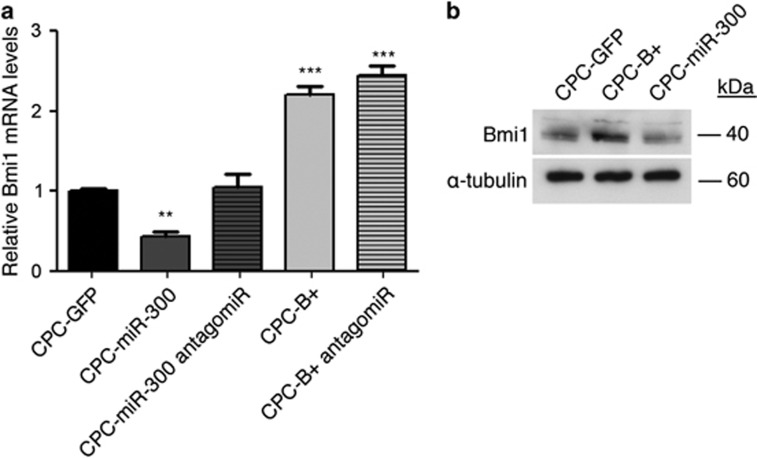
Bmi1/miR-300 negative feedback loop. (**a**) RT-qPCR analysis of Bmi1 expression in CPC-GFP, CPC-miR-300, and CPC-B+ cells, before and after treatment with the antagomiR. (**b**) Bmi1 protein levels measured by western blot; *α*-tubulin was used as a loading control. ***P<0.001, **P<0.01 (two-way ANOVA followed by Bonferroni post-test; means±S.E.M., *n*=5)

Because activation of *Bmi1* in CPCs results in increased levels of miR-300 (Figure 1), we tested whether miR-300 activation affected the levels of *Bmi1* mRNA and protein. We examined *Bmi1* expression in CPC-B+ and CPC-miR-300 cells before and after transfection with anti-miR-300. Both cell lines exhibited an increase in *Bmi1* expression following suppression of miR-300 expression (Figures 6a and b). These data suggest that Bmi1 positively regulates miR-300 and, reciprocally, miR-300 negatively regulates *Bmi1* expression in a negative feedback loop.

Taken together, our study provides evidence that miR-300 could play an important role in maintaining multipotent cell status, favouring Oct4 transcription factor expression, and negatively regulating differentiation. Since none of the confirmed genes modulated by overexpression of miR-300 or *Bmi1* (*Oct4*, *Nkx2.5, Tbx5*, and *vWF*) are predicted as direct targets (Figure 5), our results suggest a non-direct effect.

## Discussion

*Bmi1* is required for the self-renewal of adult stem cells in many tissues.^3, 6, 30, 37, 38, 39^ Because *Bmi1* knockdown reduces proliferation of mouse CPCs (Figure 1), we attempted to identify regulatory elements controlled by *Bmi1*. We used lentiviral vectors to overexpress (B+) or inhibit (sh) *Bmi1*, to study its function in CPCs. CPC-B+ cells had an enhanced proliferation capacity relative to CPC-sh-*Bmi1* cells, suggesting that *Bmi1* is important for the maintenance of some CPC primitive properties. Because miRNAs can fine tune cell homeostasis,^16, 17^ we screened for miRNAs regulated by *Bmi1*. To the best of our knowledge, all miRNAs identified in association with *Bmi1* expression are inhibitors (Supplementary Table S1). Therefore, we searched for miRNAs that could be regulated by *Bmi1* in CPCs, as plausible modulators of stem cell function.

We confirmed that miR-300 is upregulated when *Bmi1* levels are high. Also, miR-300 is highly expressed in a primary subpopulation of CPCs characterized by medium-high expression of *Bmi1*. Presumably, because of the comparatively low basal expression of miR-300 (Supplementary Figure S3b), it was not included in a recently published study on the miRNA repertoire of adult mouse CPCs.^40^ Expression of *Bmi1* in other murine cell lineages (MEFs and MSCs) consistently activated miR-300 expression, although the levels of overexpression were variable. All these results suggest that *Bmi1* regulates miR-300. BMI1 is known as a transcriptional repressor.^7^ To upregulate *c-myc* mRNA, *Bmi1* activates the Wnt pathway by repressing the Dickkopf (*Dkk*) family of Wnt inhibitors. Repression of *Dkk* genes by *Bmi1*, in particular *Dkk1,* drives the upregulation of Wnt and finally the Wnt target gene, *c-myc*.^11^ We hypothesize that a similar mechanism of suppression of a transcriptional repressor may operate to activate miR-300 when *Bmi1* is overrepresented.

*Bmi1* plays a central role in senescence and aging, regulating the expression of relevant genes involved in aging and cancer.^41, 42, 43^ Overexpression of *Bmi1* results in repression of the tumor suppressor *p16INK4a*, which has emerged as a major regulator of aging and age-associated pathologies.^44^ We show that in CPCs, *Bmi1*, and miR-300 oppositely regulate expression of *p16Ink4a* and *p19ARF*. Expression of *p16Ink4a* is decreased in CPCs upon *Bmi1* and miR-300 upregulation (Figure 2c); in contrast, *p19ARF* levels are upregulated. These two genes are localized in the same locus, sharing the common exons 2 and 3, but differing in their first exons and their respective promoters. As neither *p16Ink4a* or *p19ARF* are miR-300 targets, the present evidence suggests that the observed changes to *p16INK4a* and *p19ARF* expression are due mainly to changes in the transcriptional activity of their respective promoters. This effect on the *INK4a/ARF* locus has been also observed in cells treated with histone deacetylase inhibitors, with consequences for chromatin remodeling.^45^ It is possible that variations in BMI1 protein levels could affect PRC1 complex activity and chromatin structure; such changes seem to alter *INK4a/ARF* transcription in a way similar to histone deacetylase inhibitors. We hypothesize that the DLK1-MEG3 cluster, where miR-300 is localized, could be regulated in a similar manner.

It has previously been demonstrated that *p16Ink4a* accumulates in conjunction with p38 activation in ras-induced senescent cells, and that this accumulation is essential for senescence.^35^ Although prevention of senescence in CPC-miR-300 cells is less effective than in CPC-B+, *p16* levels are significantly reduced in CPC-miR-300 cells. This could be related to the different levels of overexpression of miR-300 in the cell populations (Supplementary Figure S3c). Thus, it is possible that miR-300 upregulation may at least partially account for the downregulation of *p16Ink4a* and the p38 pathway, thereby bypassing cell senescence. Since the most important known senescence-relevant target of Bmi1 is *p16Ink4a*, reduction of senescence in CPCs by miR-300 is almost certainly associated with reduction of *p16Ink4a* expression. In agreement with this, CPC-miR-300 cells increase the expression levels of *Oct4*. Additionally, it has been recently shown that the *Oct4/p16Ink4a* ratio directly correlates with culture life span. This result suggests that low *p16Ink4a* and high *Oct4* levels govern the senescent state of MSCs and therefore may be a good predictor of *in vitro* viability.^45^ We speculate that the same principle operates in CPCs.

Forced expression of miR-300 reduced spontaneous endothelial differentiation of CPCs, and a similar effect was obtained after *Bmi1* overexpression. Cardiac differentiation induced by retinoic acid was also reduced in CPC-miR-300 cells. These cells generated similar cardiosphere-type bodies to control cells (d0–d8), but did not differentiate further. This blockage seemed to be even stronger than that observed in CPC-B+ cells. From a panel of critical cardiogenic genes analyzed, the significant reduction found in the expression of *Nkx2.5* would be sufficient to explain the phenotype observed.^46^ Because *Nkx2.5* is not a plausible direct target for miR-300 (Figure 5), we infer that this effect is indirect.

In conclusion, miR-300 is a new member of the family of genes that control stem cell function regulated by *Bmi1* in multipotent CSCs. miR-300 favors maintenance of the undifferentiated state, inhibits differentiation, and establishes a negative feedback loop to control levels of *Bmi1*.

## Materials and Methods

### Animals

C57BL/6 mice (8–12 weeks old) were provided by Charles River Laboratories (Wilmington, MA, USA). Where indicated, Bmi1^CreER/+^; Rosa26^YFP/+^ (Bmi1-YFP) mice were generated by crossing Bmi1^CreER/+^ mice^27^ with Rosa26^YFP/+^ reporter mice (Valiente-Alendí *et al.* in press). Bmi1^CreER/+^; Rosa26^YFP/+^ double heterozygous male and female mice were injected intraperitoneally with tamoxifen (TM; Sigma; 9 mg per 40 g of body weight), every 24 h on three consecutive days between postnatal days 30 (P30) and P60. Animals were maintained and handled according to the recommendations of the CNIC Institutional Ethics Committee.

### Lentiviral construction and non-viral vectors

Lentivirus production and titration were carried out as described.^47^ Viral supernatant stocks contained 1 × 10^7^ TU/ml, with a 1:100 TU/physical particles ratio as measured by qPCR. Cells were transduced with cleared supernatants at a multiplicity of infection of 10, in 3 ml of culture medium and polybrene (Sigma) at 8 *μ*g/ml, for 8 h. Then, inoculum was removed and cell cultures were refreshed with medium before assay.

### Cellular transfection

Non-viral piggy-bac vectors (1–2 *μ*g) were co-transfected with mouse transposase-expression vector (0.5 *μ*g) in SCA1+ cells, MEFs, and MSCs, using Lipofectamine 2000 Transfection Reagent (11668-027, Sigma). MirVana miRNA inhibitor (Applied Biosystems, Carlsbad, CA, USA) was used to knockdown *mir-300* expression in a separate set of experiments. Transfection studies, in parallel with negative control miRNA inhibitor experiments, were carried out with lipofectamine RNAiMax Transfection Reagent (13778030-Sigma).

### Isolation of SCA1+ and Bmi1+ CPC cells from adult heart and cell culture

Sca1+ CPCs were isolated essentially as previously described.^48^ Enrichment for SCA1+ cells was achieved by incubating cells with a SCA1-biotinylated rat antibody (ab25196; Abcam, Cambridge, UK) and anti-rat kappa microbeads (Miltenyi Biotec, Madrid, Spain; 130-047-401), followed by purification with magnetic selection. Purified cells were plated in culture plates precoated with 0.1% gelatin and were maintained at 37 °C, 3% oxygen with the corresponding medium (IMDM, 10% FCS, 1% penicillin and streptomycin, and 1% l-glutamine, supplemented with 10 ng/ml EGF (E9644, Sigma), 20 ng/ml FGF (450-33, Pretech, London, UK), and LIF (10^3^ U; ESG1107, Millipore, Madrid, Spain). Medium was refreshed every 2 days. Cells were passaged at 70–80% confluency by trypsinization.

Bmi1+ CPC cells were obtained from hearts of Bmi1-YFP mice 5 days after TM induction. Hearts were perfused and processed as above. The resulting single-cell suspension was passed through a 40 *μ*m filter to remove debris. YFP+ cells were separated from the total heart mass with a BD FacsAria II Special Order System cell sorter fitted with a 488 nm laser to excite YFP (collected in the 525/550 channel). To discriminate YFP+ cells from autoflorescent cells, a 488 nm laser was used to excite cells, followed by collection in the 585 channel (phycoerythrin). Purified YFP+ cells were cultured at 37 ^o^C, 3% O_2_ and 5% CO_2_ in the same expansion medium used for Sca1+ cells.

### MicroRNA microarray

After SCA1+CPC transduction of the indicated lentiviral vectors (Supplementary Figure S2a), positively transduced cells were sorted, briefly expanded, and total RNA was isolated using the mirVana kit (Ambion, Madrid, Spain). RNA was quantified and integrity was checked by agarose gel electrophoresis. The total RNA required for analysis (500 ng) was prepared in 10 *μ*l RNase-free water and analyzed using Agilent miRNA microarrays (Microarray v1.0). After normalization, only those probes present in at least one sample and with average expression over the 20th percentile of all average expressions were considered for further analysis (197 miRNAs). We then used lineal models as implemented in the limma Bioconductor package.^26^ We considered the experimenter as a random variable. The analysis was performed at the CNIC Bioinformatics Unit.

### Endothelial and retinoic acid-induced cardiac differentiation

For endothelial differentiation, SCA1+ cells were cultured for 10 days in the above-indicated expansion conditions until spontaneous tubular structures appeared. For cardiac differentiation, SCA1+ cells were trypsinized and diluted to 50 000 cells/ml. Cardiospheres were prepared using hanging drop culture (20 *μ*l approx 1000 cells) for 3 days in DMEM GlutaMax supplemented with 20% FBS, 1% non-essential amino acids, and *β*-mercaptoethanol. Cardiospheres were harvested, washed, and cultured for three additional days on nonadherent Petri culture dishes (10 cm) in fresh medium. Cardiospheres were then subsequently treated with *all-trans* retinoic acid (10 nM) in DMEM GlutaMax supplemented with 15% FBS, 1% non-essential amino acids, and *β*-mercaptoetanol (50 *μ*M). The medium was replenished every 2 days.

### Apoptosis and senescence assays

To determine programmed cell death of SCA1+ cells, cells were harvested and examined for annexin V staining (APC-conjugated; 550474, BD Pharmingen, Madrid, Spain), using an apoptosis detection kit (556570 BD Pharmingen). For detection of senescence detection, cells were cultured to a density of 20 000 cells/cm^2^ in *β*-galactosidase staining buffer for 24 h (Cell Signaling Technology, Danvers, MA, USA). Cells were then fixed and stained.

### Immunofluorescence

For immunostaining, cells were fixed in 4% paraformaldehyde in PBS for 10 min at room temperature. After washing three times in PBS – 0.1% BSA for 5 min, cells were permeabilized with 0.2% Triton (Sigma, T9284) in PBS for 20 min, and washed in PBS – 0.1% BSA. Primary antibody against CD31 (MAB1398Z, Millipore) was diluted in PBS – 0.1% BSA to 1/200 and incubated overnight at 4 °C. After washing, cells were incubated with a FITC-conjugated goat anti-hamster secondary antibody (127-095-099, Jackson, Bar Harbor, ME, USA) used at a 1/1000 dilution in the presence of DAPI for 45 min (1 h at room temperature). Vectashield mounting medium without DAPI (Vectors Lab, Burlingame, CA, USA; H-1000) was applied to all slides. Fluorescent images were taken using a Nikon A1-R inverted confocal microscope.

### Gene expression analysis and western blotting

Total RNA was isolated from cultured cells using the Direct-zol RNA Miniprep Kit (Zymo, Irvine, CA, USA) and reverse transcribed with the High Capacity cDNA Reverse Transcription Kit (Applied Biosystems) for mRNA and with the TaqMan MicroRNA Reverse Transcription Kit (4366596, Applied Biosystems, Madrid, Spain) for miRNAs. Complementary DNAs were analyzed by real-time PCR using the Power SYBR Green PCR Master Mix (Applied Biosystems) for mRNA and No AmpErase TaqMan 2X Universal PCR Master Mix (S08590, Applied Biosystems) for miRNA. Amplification, detection, and data analysis were carried out with an ABI PRISM 7900HT Sequence Detection System. The crossing threshold values for individual mRNAs were normalized to *GusB* expression for mRNAs and *U6* for miRNAs. Changes in mRNA expression were denoted as the fold change relative to the control (see supplementary Table S2 for primers used).

For western blotting, total proteins were isolated using RIPA buffer (25 mM Tris–HCl pH 7.6, 150 mM NaCl, 1% NP-40, 1% sodium deoxycholate, and 0.1% SDS) and quantified by the DC Protein assay (Bio-Rad). Approximately 50 *μ*g of protein was resolved on each lane on 10% SDS-PAGE gels, electrotransferred onto nitrocellulose membrane, and probed with specific antibodies (see Supplementary Table S3 for antibodies used).

### Statistics

Data were analyzed by two-way ANOVA and Student's *t-*test. Error bars represent S.E.M. In all corresponding figures, ^∗^*P*<0.05, ^∗∗^*P*<0.01, ^∗∗∗^*P*<0.001, and ns *P*>0.05.

## Abbreviations

BMI1: B lymphoma Mo-MLV insertion region 1
CSCs/CPCs: cardiac stem/progenitor cells
GFP: green fluorescent protein
MEFs: mouse embryonic fibroblast
miR-300: microRNA miR-300
MSCs: mesenchymal stem cells
Nkx2.5: NK2 homeobox 5
Oct4: POU class 5 homeobox 1
Tbx5: T-box 5
YFP: yellow fluorescent protein
