# Supplementary material for: miR-300 mediates Bmi1 function and regulates differentiation in primitive cardiac progenitors
Source: Cell Death Dis. 2015 Oct 29;6(10):e1953–. doi: 10.1038/cddis.2015.255 (PMC4632286; doi:10.1038/cddis.2015.255)
Supplement: Supplementary Legends [file cddis2015255x1.doc]

**Supplementary Figure legends**

**Supplementary Figure S1**. (**a**) Scheme of Sca-1+ CPC isolation. (**b**) Confirmation of Sca-1+ CD45- by flow cytometry. **(c-e)** Different Sca1+ CPC gene expression profiles were measured by RT-qPCR; **(c)** Multipotent markers; **(d)** Endothelial and fibroblast markers; **(e)** Cardiac markers.

**Supplementary Figure S2**. Schematic representation of vectors and plasmids used. (**a**) Lentiviral constructs expressing Bmi1 CDS, short hairpin scramble, and short hairpin against Bmi1. (**b**) Non-viral piggy-Bac vectors to express miR-300 and GFP.

**Supplementary Figure S3**. (**a**) List of microRNAs more differentially expressed between populations. (**b**) RT-qPCR was used to confirm miRNA expression in CPC-B+ (gray bars) and CPC-shBmi1 (black bars) cells. (**c**) Relative expression of miR-300 in miR-300- (gray bar) and Bmi1- (white bar) transfected CPCs compared with control (gfp-) transfected CPCs (black bar) arbitrarily chosen as 1. ****P*<0.001, ***P*<0.01 (two-way ANOVA followed by Bonferroni post-test; means ± S.E.M., *n*= 3.

**Supplementary Figure S4.** (**a**) Cell-cycle profiles of CPC-B+, CPC-miR300, CPC-sh-Bmi1 and CPC-GFP cells were determined with propidium iodide incorporation and flow cytometry analysis. (**b**) Distribution of G_0_/G_1_, S and G_2_/M subpopulations. (**c**) Relative expression levels of *p53 and p21* in transfected cells. ns not significant; ****P*<0.001, ***P*<0.01, **P*<0.05 (two-way ANOVA followed by Bonferroni post-test; means ± S.E.M., *n*=3).

**Supplementary Figure S5.** RT-qPCR profile of miR-300 in CPC-miR300 (dark grey bars) and CPC-B+ (grey bars) cells 24 hours after transfection with mirVana miR-300 inhibitor at different concentrations. means ± S.E.M., *n*= 3.

**Supplementary Figure S6.** RT-qPCR analysis of different miR300 targets tested in CPC-GFP (black bars), CPC-miR300 (dark grey bars) and CPC-B+ (grey bars) cells. ****P*<0.001 two-way ANOVA followed by Bonferroni post-test; means ± S.E.M., *n*=5).

**Supplementary Figure S7**. **CPC-miR-300 vs CPC-Bmi1.** Main common and differential cellular parameters.

**Supplementary Table S1. miRNAs regulating Bmi-1 and supplementary references**

**Supplementary Table S2. RT-qPCR primers**

**Supplementary Table S3. Antibodies**
