# Supplementary figures and images for: miR-300 mediates Bmi1 function and regulates differentiation in primitive cardiac progenitors
Source: Cell Death Dis. 2015 Oct 29;6(10):e1953–. doi: 10.1038/cddis.2015.255 (PMC4632286; doi:10.1038/cddis.2015.255)

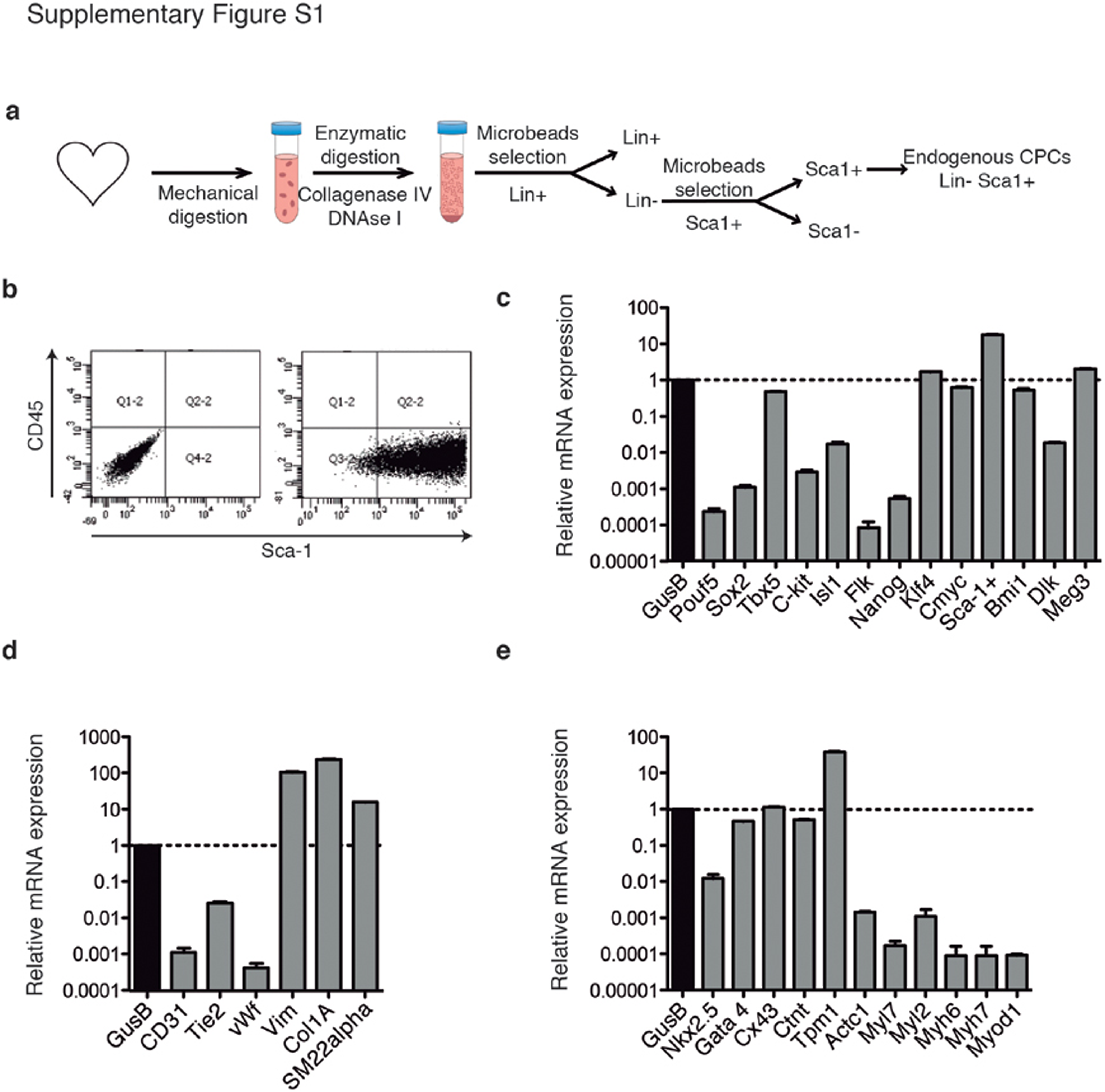

Supplement: Supplementary Figure 1 [file cddis2015255x2.tif]

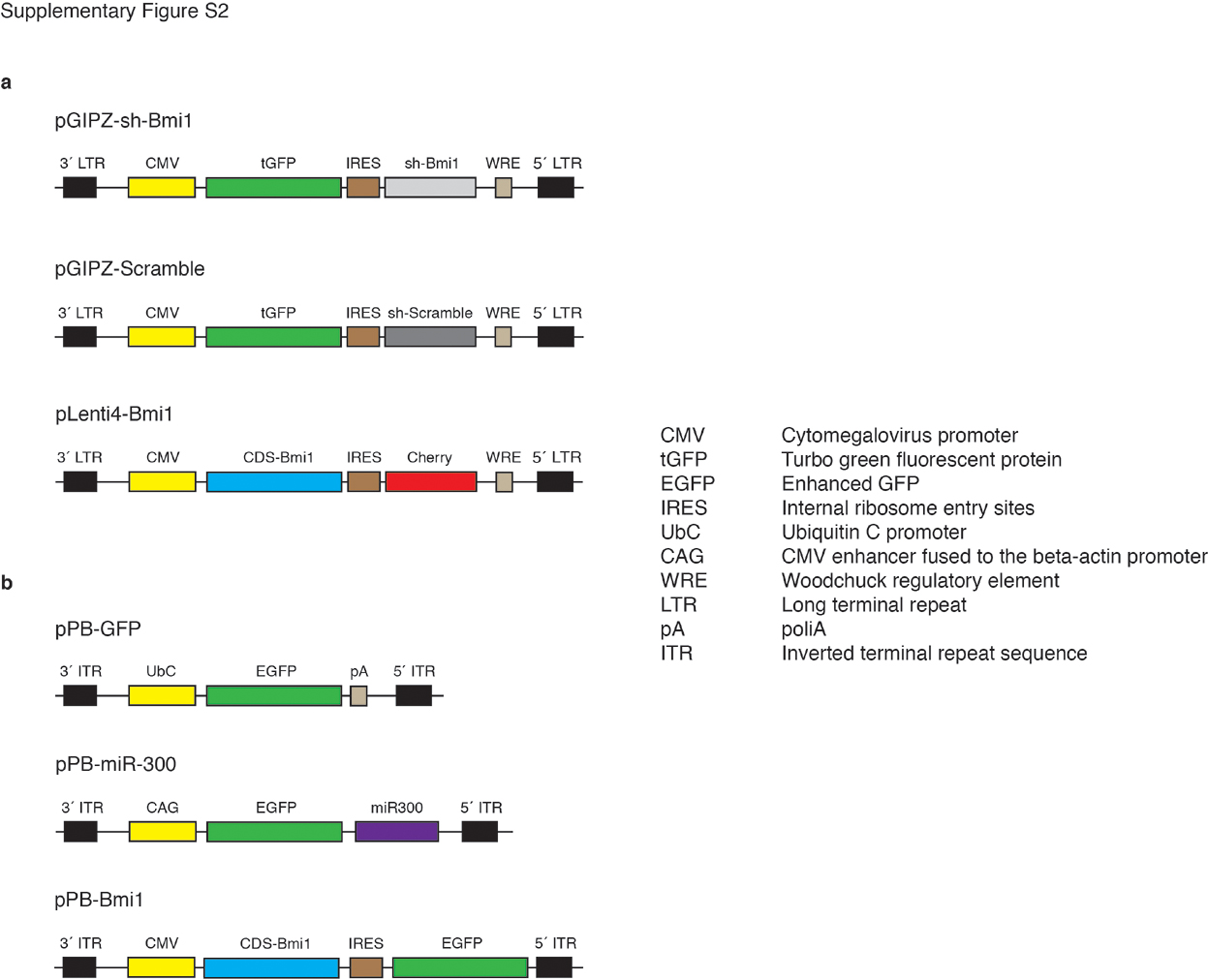

Supplement: Supplementary Figure 2 [file cddis2015255x3.tif]

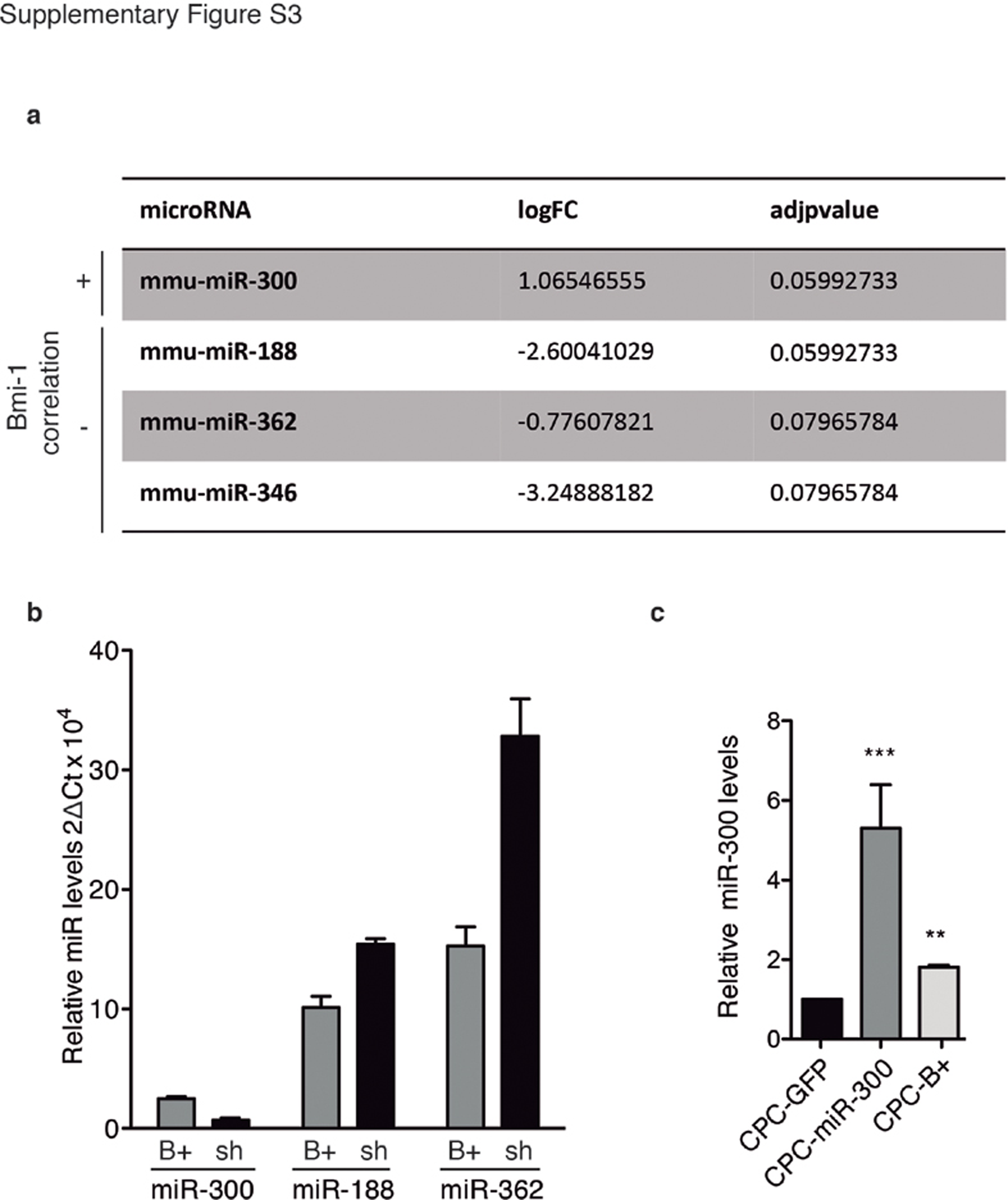

Supplement: Supplementary Figure 3 [file cddis2015255x4.tif]

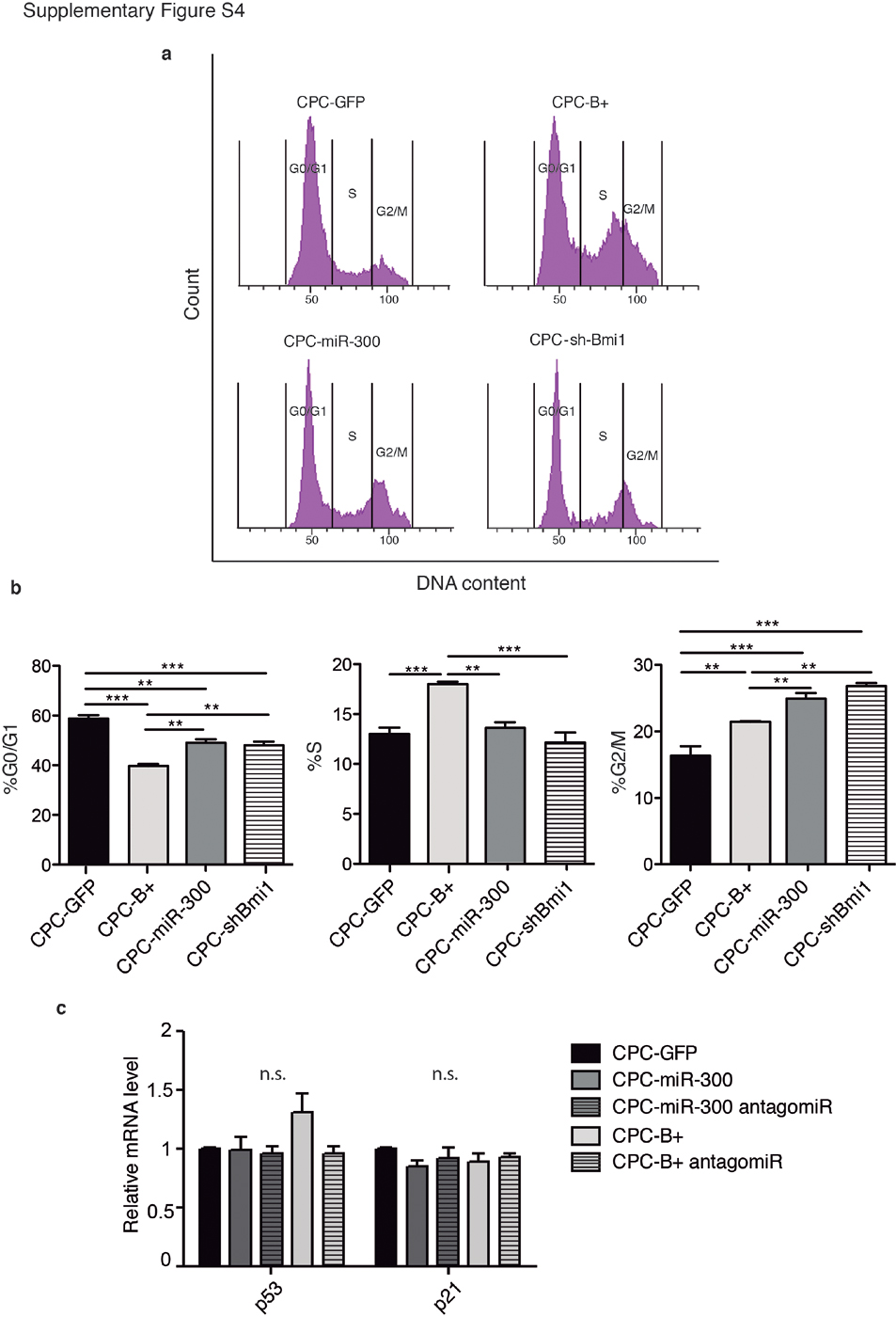

Supplement: Supplementary Figure 4 [file cddis2015255x5.tif]

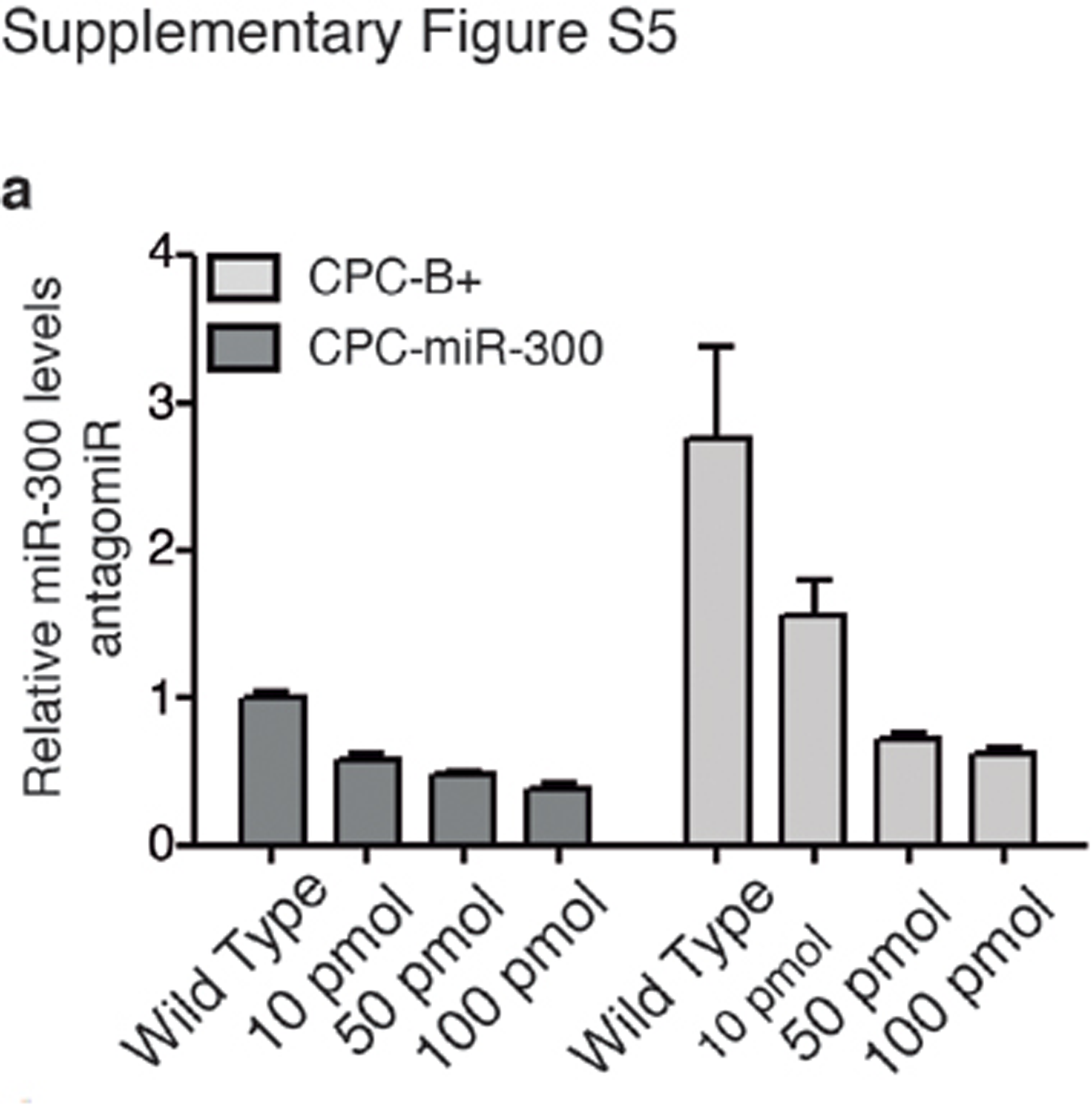

Supplement: Supplementary Figure 5 [file cddis2015255x6.tif]

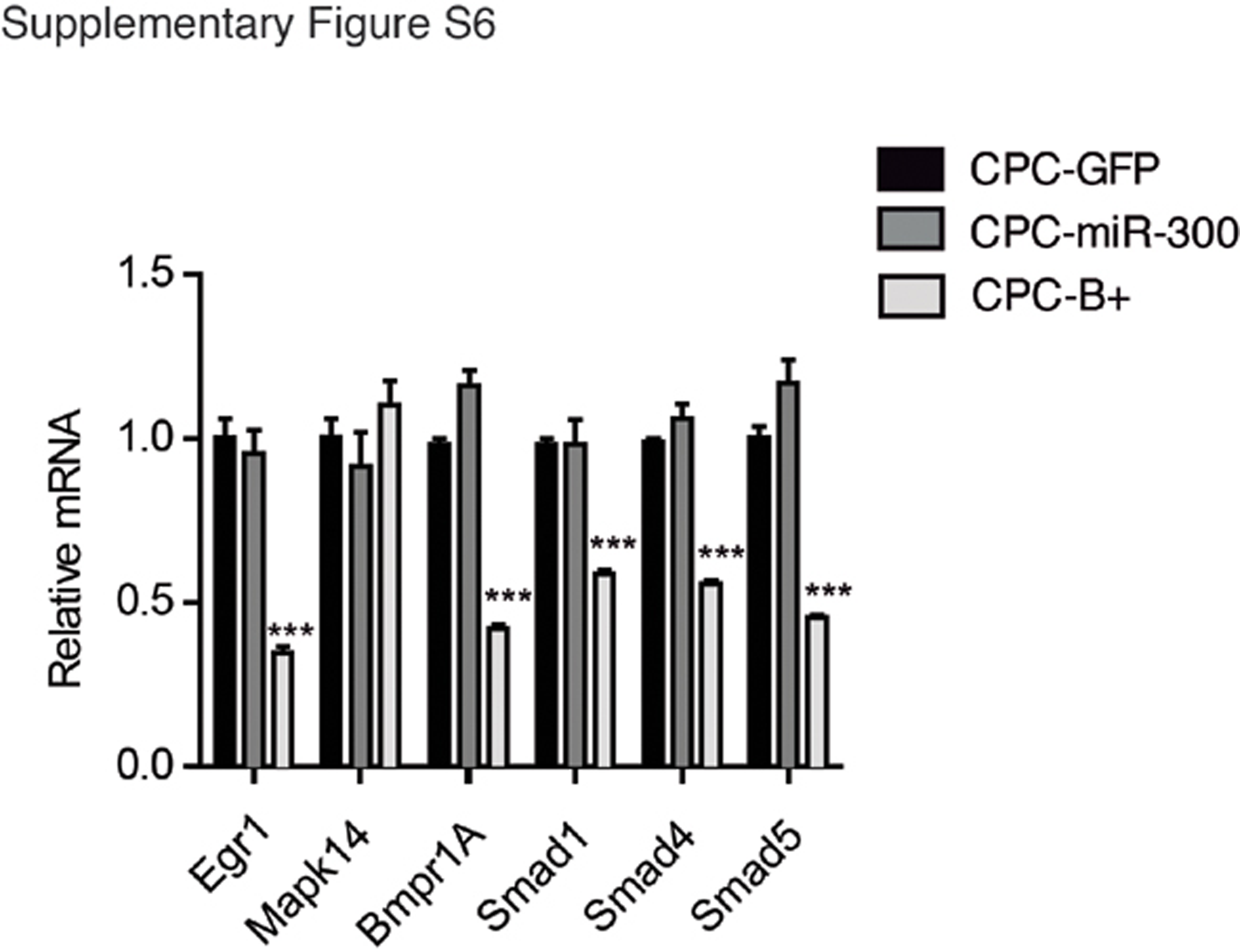

Supplement: Supplementary Figure 6 [file cddis2015255x7.tif]

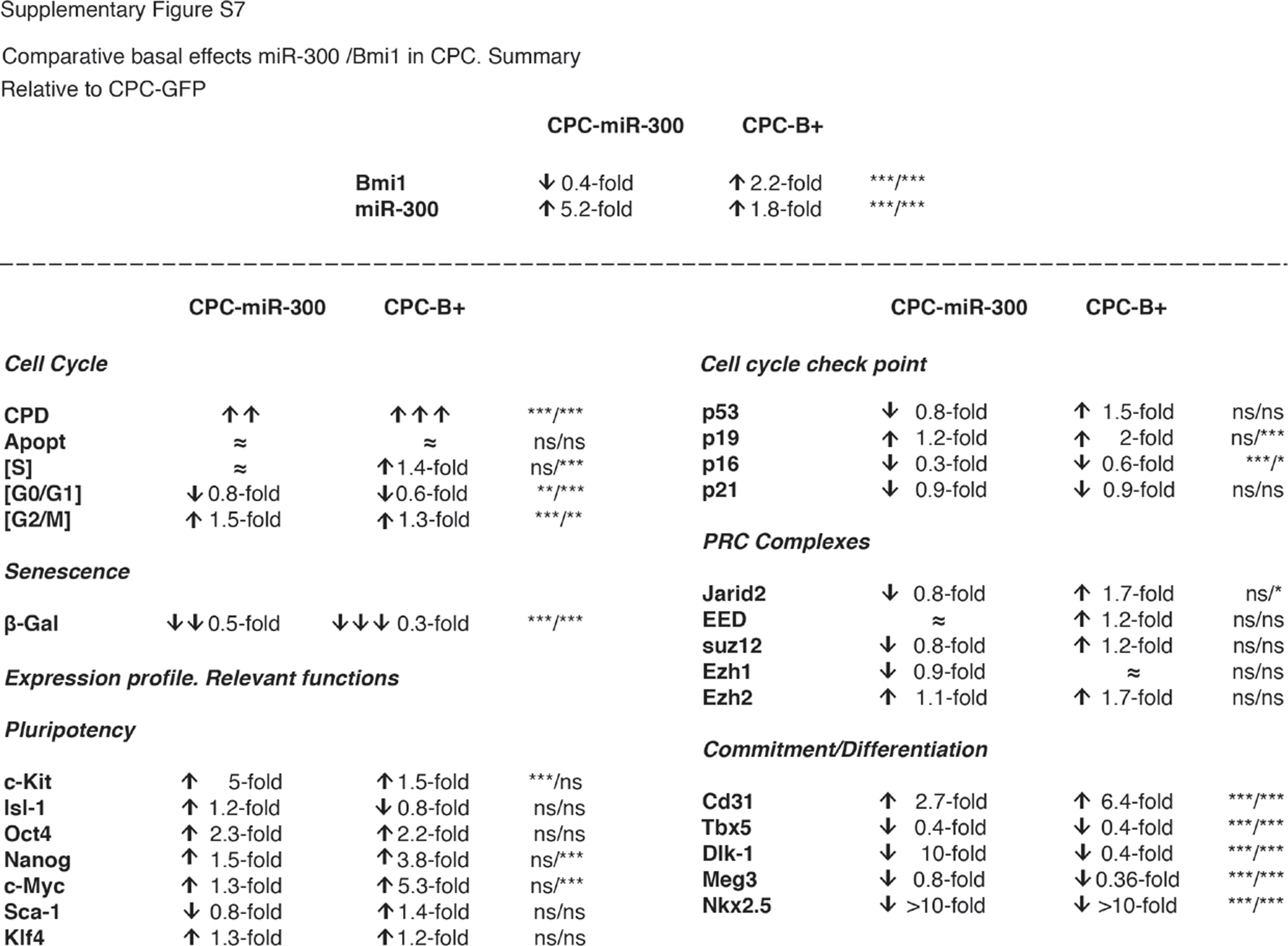

Supplement: Supplementary Figure 7 [file cddis2015255x8.tif]
