# Supplementary material for: miR-300 mediates Bmi1 function and regulates differentiation in primitive cardiac progenitors
Source: Cell Death Dis. 2015 Oct 29;6(10):e1953–. doi: 10.1038/cddis.2015.255 (PMC4632286; doi:10.1038/cddis.2015.255)
Supplement: Supplementary Table 1 [file cddis2015255x9.doc]

**Supplementary Table S1**

miRNAs regulating Bmi1

miR-203

miR-200c

miR-218

miR-200b

miR-34

miR-194

miR-300

miR-494

miR-495

miR-539

miR-543

miR-544

miR-128

miR-141

miR-16

miR-15

miR-26b

miR-135a

miR-487b

Pathological process

Epithelial-mesenchymal transition/Breast c.

Breast/Head and neck squamous carcinoma/

Melanoma/Epithelial-mesenchymal transitions

Colon cancer/Melanoma

Epithelial-mesenchymal transition

Epithelial-mesenchymal transition

Epithelial-mesenchymal transition

Epithelial-mesenchymal transition

Epithelial-mesenchymal transition

Epithelial-mesenchymal transition

Epithelial-mesenchymal transition

Epithelial-mesenchymal transition

Epithelial-mesenchymal transition

Glioblastoma/Medulloblastoma/Pituitary

Senescence of human diploid fibroblasts

Ovarian/Hepatocellular carcinoma

Ovarian/Hepatocellular carcinoma/

Pituitary cancer

Pancreatic ductal adenocarcinoma

Pulmonary carcinogenesis

References

1, 2

1- 4

5- 7

7, 8

9

10

11

12

12

12

12

12

12

13- 16

17

18

18, 19

15

20

21

**REFERENCES**

1. Wellner U, Schubert J, Burk UC, Schmalhofer O, Zhu F, Sonntag A *et al.* The EMT-activator ZEB1 promotes tumorigenicity by repressing stemness-inhibiting microRNAs. *Nature cell biology* 2009; **11**(12)**:** 1487-95.

2. Yin J, Zheng G, Jia X, Zhang Z, Zhang W, Song Y *et al.* A Bmi1-miRNAs cross-talk modulates chemotherapy response to 5-fluorouracil in breast cancer cells. *PloS one* 2013; **8**(9)**:** e73268.

3. Shimono Y, Zabala M, Cho RW, Lobo N, Dalerba P, Qian D *et al.* Downregulation of miRNA-200c links breast cancer stem cells with normal stem cells. *Cell* 2009; **138**(3)**:** 592-603.

4. Lo WL, Yu CC, Chiou GY, Chen YW, Huang PI, Chien CS *et al.* MicroRNA-200c attenuates tumour growth and metastasis of presumptive head and neck squamous cell carcinoma stem cells. *J Pathol* 2011; **223**(4)**:** 482-95.

5. Liu S, Tetzlaff MT, Cui R, Xu X. miR-200c inhibits melanoma progression and drug resistance through down-regulation of BMI-1. *Am J Pathol* 2012; **181**(5)**:** 1823-35.

6. Kopp F, Oak PS, Wagner E, Roidl A. miR-200c sensitizes breast cancer cells to doxorubicin treatment by decreasing TrkB and Bmi1 expression. *PloS one* 2012; **7**(11)**:** e50469.

7. He X, Dong Y, Wu CW, Zhao Z, Ng SS, Chan FK *et al.* MicroRNA-218 inhibits cell cycle progression and promotes apoptosis in colon cancer by downregulating BMI1 polycomb ring finger oncogene. *Mol Med* 2012; **18:** 1491-8.

8. Wei Y, Du Y, Chen X, Li P, Wang Y, Zang W *et al.* Expression patterns of microRNA-218 and its potential functions by targeting CIP2A and BMI1 genes in melanoma. *Tumour Biol* 2014; **35**(8)**:** 8007-15.

9. Sun L, Yao Y, Liu B, Lin Z, Lin L, Yang M *et al.* MiR-200b and miR-15b regulate chemotherapy-induced epithelial-mesenchymal transition in human tongue cancer cells by targeting BMI1. *Oncogene* 2012; **31**(4)**:** 432-45.

10. Siemens H, Jackstadt R, Hunten S, Kaller M, Menssen A, Gotz U *et al.* miR-34 and SNAIL form a double-negative feedback loop to regulate epithelial-mesenchymal transitions. *Cell cycle* 2011; **10**(24)**:** 4256-71.

11. Dong P, Kaneuchi M, Watari H, Hamada J, Sudo S, Ju J *et al.* MicroRNA-194 inhibits epithelial to mesenchymal transition of endometrial cancer cells by targeting oncogene BMI-1. *Molecular cancer* 2011; **10:** 99.

12. Haga CL, Phinney DG. MicroRNAs in the imprinted DLK1-DIO3 region repress the epithelial-to-mesenchymal transition by targeting the TWIST1 protein signaling network. *The Journal of biological chemistry* 2012; **287**(51)**:** 42695-707.

13. Cui JG, Zhao Y, Sethi P, Li YY, Mahta A, Culicchia F *et al.* Micro-RNA-128 (miRNA-128) down-regulation in glioblastoma targets ARP5 (ANGPTL6), Bmi-1 and E2F-3a, key regulators of brain cell proliferation. *J Neurooncol* 2010; **98**(3)**:** 297-304.

14. Venkataraman S, Alimova I, Fan R, Harris P, Foreman N, Vibhakar R. MicroRNA 128a increases intracellular ROS level by targeting Bmi-1 and inhibits medulloblastoma cancer cell growth by promoting senescence. *PloS one* 2010; **5**(6)**:** e10748.

15. Palumbo T, Faucz FR, Azevedo M, Xekouki P, Iliopoulos D, Stratakis CA. Functional screen analysis reveals miR-26b and miR-128 as central regulators of pituitary somatomammotrophic tumor growth through activation of the PTEN-AKT pathway. *Oncogene* 2013; **32**(13)**:** 1651-9.

16. Peruzzi P, Bronisz A, Nowicki MO, Wang Y, Ogawa D, Price R *et al.* MicroRNA-128 coordinately targets Polycomb Repressor Complexes in glioma stem cells. *Neuro Oncol* 2013; **15**(9)**:** 1212-24.

17. Dimri M, Carroll JD, Cho JH, Dimri GP. microRNA-141 regulates BMI1 expression and induces senescence in human diploid fibroblasts. *Cell cycle* 2013; **12**(22)**:** 3537-46.

18. Bhattacharya R, Nicoloso M, Arvizo R, Wang E, Cortez A, Rossi S *et al.* MiR-15a and MiR-16 control Bmi-1 expression in ovarian cancer. *Cancer research* 2009; **69**(23)**:** 9090-5.

19. Sun L, Zhang D, Liu F, Xiang X, Ling G, Xiao L *et al.* Low-dose paclitaxel ameliorates fibrosis in the remnant kidney model by down-regulating miR-192. *J Pathol* 2011; **225**(3)**:** 364-77.

20. Dang Z, Xu WH, Lu P, Wu N, Liu J, Ruan B *et al.* MicroRNA-135a inhibits cell proliferation by targeting Bmi1 in pancreatic ductal adenocarcinoma. *Int J Biol Sci* 2014; **10**(7)**:** 733-45.

21. Xi S, Xu H, Shan J, Tao Y, Hong JA, Inchauste S *et al.* Cigarette smoke mediates epigenetic repression of miR-487b during pulmonary carcinogenesis. *The Journal of clinical investigation* 2013; **123**(3)**:** 1241-61.
