# Supplementary material for: miR-300 mediates Bmi1 function and regulates differentiation in primitive cardiac progenitors
Source: Cell Death Dis. 2015 Oct 29;6(10):e1953–. doi: 10.1038/cddis.2015.255 (PMC4632286; doi:10.1038/cddis.2015.255)
Supplement: Supplementary Table 2 [file cddis2015255x10.doc]

**Supplementary Table S2**

Primers

1. miRNAs

| microRNAs | Reference |
| --- | --- |
| miR-188 | 002320 Applied Biosystems |
| miR-300 | 000191Applied Biosystems |
| miR-346 | 001064 Applied Biosystems |
| miR-362 | 002614 Applied Biosystems |
| miR-376b | 002452 Applied Biosystems |
| miR-381 | 000571 Applied Biosystems |
| miR-654 | 002522 Applied Biosystems |
| U6 snRNA | 001973 Applied Biosystems |

1. mRNAs

| Gene | Forward | Reverse |
| --- | --- | --- |
| Actc1 | GCTCTGGGCTTCATCACCTA | AGCTGTCTTCCCGTCCATC |
| Bmi1 | TTTTATGCTGAACGACTTTTAACTT | GCTCAGTGATCTTGATTCTGGT |
| Bmpr1A | TTTATGGCACCCAAGGAAAG | TGGTATTCAAGGGCACATCA |
| c-kit | ATTGTGCTGGATGGATGGAT | GATCTGCTCTGCGTCCTGTT |
| c-myc | TGAAGTTCACGTTGAGGGG | AGAGCTCCTCGAGCTGTTTG |
| CD31 | AGTTGCTGCCCATTCATCAC | CTGGTGCTCTATGCAAGCCT |
| Col1A | TAGGCCATTGTGTATGCAGC | ACATGTTCAGCTTTGTGGACC |
| cTNT | AGTTTAAGCGGCCCACCCTC | TTCTTGCGCCAGTCTCCCAC |
| Cx43 | GGACCTTGTCCAGCAGCTT | TCCAAGGAGTTCCACCACTT |
| Dlk1 | TGTGCAGGAGCATTCGTACT | CGGGAAATTCTGCGAAATAG |
| EED | CAACACCAGCCACCCTCTAT | GAGAAGGTTTGGGTCTCGTG |
| Ezh1 | ACCTGCTGCTGGCCCCTTC | GACTCCTTGATGAAGGTGCCC |
| Ezh2 | GACAGTGACAGAGAAGCAGGG | ATCCTCAGTGGGAACAGGTGC |
| Fgf1r | ATGGTTGACCGTTCTGGAAG | GGAAGTCGCTCTTCTTGGTG |
| Flk | TCCAGAATCCTCTTCCATGC | AAACCTCCTGCAAGCAAATG |
| Gata4 | CCATCTCGCCTCCAGAGT | CTGGAAGACACCCCAATCTC |
| GusB | ACTCCTCACTGAACATGCGA | ATAAGACGCATCAGAAGCCG |
| Igf1r | CAAGCTGTGTGTCTCCGAAA | TGATTCGGTTCTTCCAGGTC |
| Islt1 | CACGAAGTCGTTCTTGCTGA | GGTTAGGGATGGGAAAACCT |
| Jarid2 | GCAAGGAAAGACCCAAGAGG | CAAGGCCTTCCCCATGCTGC |
| Klf4 | CAGTGGTAAGGTTTCTCGCC | GCCACCCACACTTGTGACTA |
| Map2k3 | CACGGTCGACTGCTTCTACA | AGGACATTGGATGGCTTCAC |
| Mapk14 | ATCATTCACGCCAAAAGGAC | AGCTTCTGGCACTTCACGAT |
| Mef2a | GAGCCTCATGAAAGCAGGAC | GAAGTTCTGAGGTGGCAAGC |
| Meg3 | TTACAGTTGGAGGGTCCTGG | CGAGGACTTCACGCACAAC |
| Myh7 | GAGCCTTGGATTCTCAAACG | GTGGCTCCGAGAAAGGAAG |
| Myl2 | TGGGTAATGATGTGGACCAA | GGGAGGTTCTCCAAAGAGGA |
| Myl7 | CTCTTCCTTGTTCACCACCC | CTCACACTCTTCGGGGAGAA |
| Myod1 | TTAAGCCTTGGTTAGCCAGC | GGGGTCTGAACACTCTTTGC |
| Nanog | CTGAGATGCTCTGCACAGAGG | GGTCTTAACCTGCTTATAGCTC |
| Nkx2.5 | TGGTTGGAGGTGACTTTGTG | GGCAGTGATGACCTGGAGTT |
| p16 | CGTACCCCGATTCAGGTGATG | AGAAGGTAGTGGGGTCCTCG |
| p19 | GCAGAAGAGCTGCTACGTGA | CGTGAACATGTTGTTGAGGC |
| p21 | ATCACCAGGATTGGACATGG | CGGTGTCAGAGTCTAGGGGA |
| p53 | TCCGACTGTGACTCCTCCAT | CTAGCATTCAGGCCCTCATC |
| Pouf5f1 | TCTTCTGCTTCAGCAGCTTG | GTTGGAGAAGGTGGAACCAA |
| Sca1 | GGTTCTTTAGGCTGGCAGTG | GGGAAGTTTCCATGGTGAAG |
| SM22alpha | CCTCCAGCTCCTCGTCATAC | CCTTCCAGTCCACAAACGAC |
| Smad1 | CAGCAGCTACCCCAACTCTC | CCAGTGTTTTGGTTCCTCGT |
| Smad4 | CCTGTTGTGACTGTGGATGG | CCAAACGTCACCTTCACCTT |
| Smad5 | TAGGCGGCATATTGGAAAAG | CCTCGAATCCATGGTTGACT |
| Sox2 | AAAGCGTTAATTTGGATGGG | ACAAGAGAATTGGGAGGGGT |
| suz12 | TACCCTGGAAGTCCTGCTTG | AACTGCCAGGGATGGAAAAT |
| Tbx5 | GGCTTTGTCCAGCTCCACT | ATTTTACCCGGGAGCCTAC |
| Tie2 | TTTCGGCATCAGACACAAGA | CCGGCTTAGTTCTCTGTGGA |
| Tpm1 | TCCAACTCCTCCTCAACCAG | CTCAAAGATGCCCAGGAGAA |
| Vim | TCCACTTTCCGTTCAAGGTC | AGAGAGAGGAAGCCGAAAGC |
| VWF | CCGTCTTCAGTAGCTGGCAT | GTGTAAACGGGCATCTCCTC |
| αMHC | GGCCACATCCGTGCAGATAG | TCCAGCTTCACGCGGTACTC |
