# Supplementary material for: miR-300 mediates Bmi1 function and regulates differentiation in primitive cardiac progenitors
Source: Cell Death Dis. 2015 Oct 29;6(10):e1953–. doi: 10.1038/cddis.2015.255 (PMC4632286; doi:10.1038/cddis.2015.255)
Supplement: Supplementary Table 3 [file cddis2015255x11.doc]

**Supplementary Table S3**

Antibodies

Bmi1 western 1/200 Abcam ab35842

Nkx2.5 western 1/500 Abcam ab35842

α-tubulin western 1/1000 Sigma-Aldrich T9026

SCA1-biot puri 1/100 Abcam ab25196

CD31 IMM 1/200 Millipore MAB1398Z
